# Handgrip strength as a valuable multidimensional clinical parameter: a cross-sectional Scopus-based analysis

**DOI:** 10.1007/s00296-026-06157-6

**Published:** 2026-05-28

**Authors:** Umida Khojakulova, Dinara Makhanbetkulova, Bekzhan A. Permenov, Yuliya Fedorchenko, Burhan Fatih Kocyigit

**Affiliations:** 1 Department of Emergency Medicine and Nursing, South Kazakhstan Medical Academy, Shymkent, Kazakhstan; 2 Department of Nursing, Asfendiyarov Kazakh National Medical University, Almaty, Kazakhstan; 3 Department of Cardiac Surgery Anesthesiology and Intensive Care, Heart Center Shymkent, Shymkent, Kazakhstan; 4 Department of Social Health Insurance and Public Health, South Kazakhstan Medical Academy, Shymkent, Kazakhstan; 5 Department of Internal Medicine, Khoja Akhmet Yassawi International Kazakh-Turkish University, Turkistan, Kazakhstan; 6 Department of Pathophysiology, Ivano-Frankivsk National Medical University, Ivano- Frankivs’k, Ukraine; 7 Department of Physical Medicine and Rehabilitation, University of Health Sciences, Adana City Research and Training Hospital, Adana, Türkiye

**Keywords:** Hand strength, Muscle strength, Sarcopenia, Bibliometrics, Databases, Bibliographic

## Abstract

This study aims to evaluate the bibliometric profile of handgrip strength (HGS) research using the Scopus database. A comprehensive search of the Scopus database was conducted on April 26, 2026. No temporal restrictions were imposed, and all publication types from 1944 to 2026 were included in the analysis. Publication trends over time were assessed using linear regression analysis. Distributions by country, institution, journal, author, funding source, and keywords were examined. The ten most cited publications were identified, and their bibliographic data were recorded. Countries contributing at least 1% of the total publications were designated as main active countries. Retracted publications in the dataset were categorized using a predetermined system based on the reasons provided in the retraction notifications. A total of 12,221 publications were included in the analysis. The literature, originating in 1944, reached its peak in 2025 with 1,553 publications. Linear regression analysis demonstrated a statistically significant upward trend (B = 8.76; R² = 0.417; *p* < 0.001). Of the 128 countries contributing, the United States (*n* = 1979), Brazil (*n* = 1577), and China (*n* = 1115) accounted for the highest publication counts. The most prolific journals were *Nutrients* (*n* = 273), *International Journal of Environmental Research and Public Health* (*n* = 241), and *PLOS One* (*n* = 223). The most frequently cited studies primarily addressed the diagnosis and treatment of sarcopenia. The most productive institution was Universidade de São Paulo (*n* = 273), and the most prolific author was Izquierdo, M. (*n* = 74). Six publications were retracted, published between 2021 and 2023. The bibliometric analysis demonstrates a statistically significant upward trend in HGS research spanning more than eight decades. Contributions from 128 countries highlight the global scientific engagement in this field. The predominance of sarcopenia-related studies among the most-cited publications underscores the central role of musculoskeletal research in establishing HGS as a clinically recognized marker.

## Introduction

Handgrip strength (HGS), indexed in the Medical Subject Headings (MeSH) database under the descriptor “Hand Strength”, is defined as the maximal voluntary force produced by the hand and forearm muscles during a gripping maneuver [1]. Due to its simplicity, reproducibility, and minimal equipment requirements, HGS measurement is widely recognized as an objective indicator of muscular performance in clinical medicine [2, 3].

HGS measurement with handheld dynamometers is rapid, cost-effective, and easily standardized, making it well-suited for routine clinical screening [4, 5]. A primary clinical application of HGS assessment is the evaluation of sarcopenia, a progressive skeletal muscle disorder characterized by loss of muscle strength and mass, particularly in older adults [6, 7]. There is increasing interest in integrating digital health applications, remote monitoring technologies, and smart dynamometers into HGS assessment. These innovations support continuous, real-time monitoring of muscle strength in clinical and home settings, improving the feasibility of large-scale studies and remote patient follow-up [8].

The Asian Working Group for Sarcopenia established sex-specific criteria for low HGS, defining values below 26 kg in men and 18 kg in women as indicative of reduced muscle function, based on the lower 20th percentile of the reference population [9]. In contrast, the European Working Group on Sarcopenia in Older People recommended slightly different thresholds, setting HGS cut-off values at 27 kg for men and 16 kg for women, which reflect regional differences in normative data and diagnostic methodologies [9].

Beyond its established role in geriatric medicine, HGS demonstrates significant clinical utility across multiple medical disciplines [10–14]. In cardiology, reduced grip strength is associated with increased cardiovascular morbidity and mortality [10]. In rheumatology, HGS functions as a reliable indicator of musculoskeletal impairment and disease burden in inflammatory joint disorders [11, 12]. Recent studies in endocrinology indicate potential associations between HGS and hormonal status [13, 14]. In reproductive health, an extended reproductive lifespan correlates with a lower risk of reduced HGS in postmenopausal women [15]. Within orthopedics and metabolic bone disease, low HGS has been recognized as an independent predictor of decreased bone mineral density and increased risk of fragility fractures in postmenopausal women [16].

The increasing recognition of HGS as a clinically significant and prognostically valuable marker has coincided with a substantial rise in scientific publications exploring its methodological, clinical, and epidemiological dimensions. Despite this growth, no comprehensive bibliometric analysis has yet mapped the global research landscape of HGS literature. Within this context, bibliometric analysis offers a systematic framework for evaluating the development of research activity in the field. By incorporating quantitative metrics, such as publication volume and country-based data, bibliometric methods facilitate the identification of influential studies, prominent research institutions, prolific authors, and emerging research themes. Previous publications have primarily examined specific clinical applications or population subgroups, resulting in a limited understanding of broader publication trends, key contributors, and collaborative networks. This study seeks to fill this gap by presenting a comprehensive bibliometric analysis of the global HGS literature, thereby establishing a basis for future research prioritization and fostering international collaboration.

## Methods

### Search setting

A search of the Scopus database was conducted on April 26, 2026, in accordance with established recommendations to ensure comprehensive coverage of the literature for bibliometric analyses [17]. The search strategy targeted publications concerning HGS, as indexed in the Medical Subject Headings (MeSH) database under the descriptor “Hand Strength”. The keyword “handgrip strength” was applied to the TITLE, ABSTRACT, and/or KEYWORDS fields (TITLE–ABSTRACT–KEYWORDS) in the Scopus advanced search interface to identify relevant records for analysis. Scopus was chosen as the primary data source due to its broad coverage of peer-reviewed scientific literature and its integrated analytical tools for evaluating large bibliographic datasets [18].

No temporal restrictions were imposed on the literature search; therefore, all publications indexed in the Scopus database from the earliest record in 1944 through 2026 were eligible for inclusion. This approach was adopted to provide a comprehensive historical overview of the field’s evolution and to capture the complete trajectory of scientific output over more than eight decades. Furthermore, no limitations were imposed on document type; all publication categories available in Scopus, including original articles, reviews, conference papers, letters, editorials, book chapters, and other document types, were included in the analysis. To ensure consistency and comparability of bibliometric indicators across studies, only English-language publications were retained in the final dataset [19].

### Data collection

All documents retrieved from the Scopus database that satisfied the predefined search criteria were systematically recorded and analyzed using bibliometric methods. The annual distribution of publications was assessed from 1944 to 2026. Subsequently, the geographic distribution of publications was evaluated to identify the total number of contributing countries. To differentiate the most active countries from those with limited output, countries that contributed at least 1% of total publications were designated as “main active countries " [20, 21]. The five most productive journals in the field were identified according to total publication output. The distribution of documents by publication type was analyzed, and publication counts for each category were recorded. The ten most prolific institutions were determined based on their total number of publications. The ten most productive authors were also identified, with individual publication counts documented. The five most influential funding sponsors were determined by the number of publications they supported. The twenty most frequently keywords were identified. The ten most cited publications in HGS research were identified, and key bibliographic details, including title, authors, journal, publication year, and total citations, were extracted and recorded for each publication.

Retracted publications in the dataset were reviewed to determine their scope and reasons for retraction. For each article, data collected included title, journal, publication year, citation count, and the corresponding author’s country. Retraction reasons were classified using a predetermined system based on those explicitly stated in the retraction notices [22, 23]. Each notice was independently assessed, and the reasons were categorized. If a publication listed multiple justifications, each was coded separately, allowing for multiple classifications per retraction.

All findings and rankings in this study are based solely on data from the Scopus database and are limited to English-language publications.

### Statistical analyses

Descriptive data were reported as numbers (n) and percentages (%). Temporal trends in annual publication output were assessed using linear regression in the Statistical Package for the Social Sciences (SPSS) version 26.0 (SPSS Inc., Chicago, IL, USA). Statistical significance was set at *p* < 0.05.

## Results

**Fig. 1:**
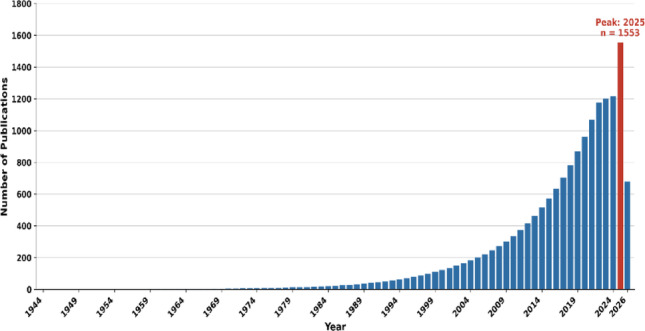
Temporal distribution of annual publications from 1944 to 2026

A comprehensive bibliometric analysis was conducted on 12,221 documents identified through the search strategy outlined in the Methods section. The earliest publication in this field appeared in 1944. Analysis of annual publication numbers revealed a marked increase, with the highest number in 2025 (*n* = 1,553). To assess the magnitude and direction of this trend, a linear regression was performed with publication year as the independent variable. The model was statistically significant (R² = 0.417, *p* < 0.001), indicating that publication year was a significant positive predictor of annual output (B = 8.76, 95% CI 6.46–11.06; t = 7.61, *p* < 0.001). These findings demonstrate that publication volume increased by approximately 8.76 articles per year throughout the study period, providing clear statistical evidence of a sustained upward trend in scientific productivity in this domain (Fig. 1).

Analysis of publication output by country identified contributions from 128 countries. To determine the most scientifically active nations, a threshold of 1% of the total 12,221 publications was established. Countries meeting or exceeding this threshold were designated as main active countries, resulting in 30 countries classified in this category (Table 1). The United States (*n* = 1979; 16.19%), Brazil (*n* = 1,577; 12.9%), and China (*n* = 1,115; 9.12%) were the top three contributors, together accounting for over one-third of the global output. Singapore, at the lower threshold, contributed 127 publications (1.04%), representing the minimum for inclusion as a main active country.

**Table 1:** The main active countries in handgrip strength research

Rank	Country	Publications	% of total publications
1	United States	1979	16.19%
2	Brazil	1577	12.90%
3	China	1115	9.12%
4	Spain	1053	8.62%
5	Japan	947	7.75%
6	United Kingdom	886	7.25%
7	Italy	759	6.21%
8	Canada	653	5.34%
9	Australia	569	4.66%
10	South Korea	563	4.61%
11	Türkiye	500	4.09%
12	Netherlands	486	3.98%
13	Germany	435	3.56%
14	Portugal	370	3.03%
15	France	348	2.85%
16	Sweden	331	2.71%
17	Chile	306	5%
18	Denmark	282	2.31%
19	India	281	3%
20	Taiwan	279	2.28%
21	Belgium	224	1.83%
22	Poland	222	1.82%
23	Switzerland	217	1.78%
24	Greece	172	1.41%
25	Colombia	146	1.19%
26	Iran	144	1.18%
27	Malaysia	143	1.17%
28	Finland	143	1.17%
29	Austria	134	.1%
30	Singapore	127	1.04%

All data are derived from the Scopus database.

**Fig. 2:**
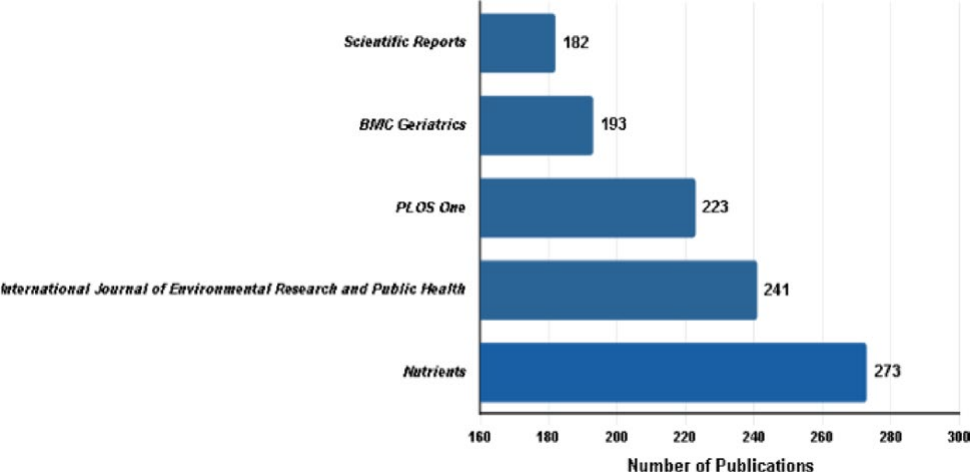
The five most productive journals in handgrip strength research by number of publications

The leading journals in this field, based on publication volume, were *Nutrients* (*n* = 273), *International Journal of Environmental Research* and *Public Health* (*n* = 241), *PLOS One* (*n* = 223), *BMC Geriatrics* (*n* = 193), and *Scientific Reports* (*n* = 182) (Fig. 2).

**Fig. 3:**
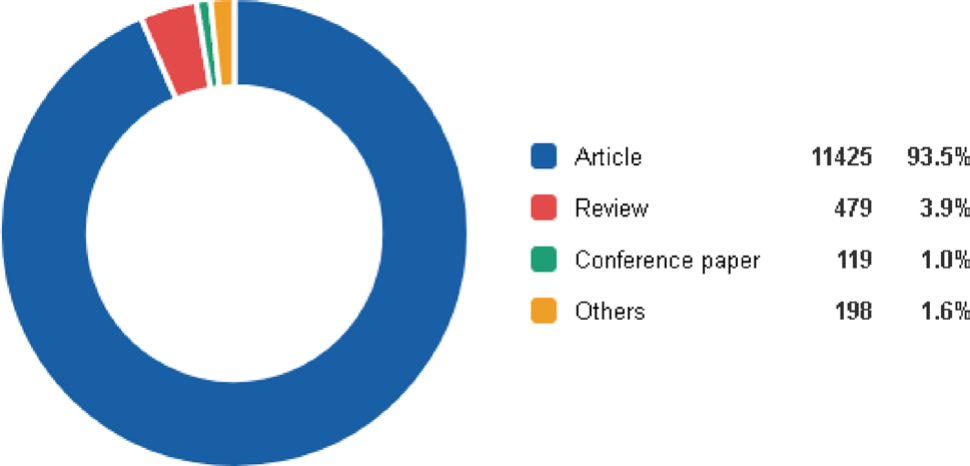
Distribution of document types

Total of 12,221 documents were retrieved across 12 identified publication types. Original articles constituted the majority of the literature (*n* = 11,425; 93.5%), followed by reviews (*n* = 479; 3.9%) and conference papers (*n* = 119; 1%). The remaining publication types, including letters, book chapters, editorials, and errata, were consolidated into a single “others” category, which accounted for 198 documents (1.6%) of the overall output (Fig. 3).

The ten most productive institutions collectively accounted for 1,390 publications. Universidade de São Paulo (Brazil) led with 273 publications, followed by Universidad de Granada (Spain, *n* = 202) and Karolinska Institutet (Sweden, *n* = 175) (Table 2). At the national level, Spain exhibited the highest institutional representation in this group, with three institutions: Universidad de Granada, Instituto de Salud Carlos III, and Universidad Pública de Navarra. Brazil contributed two institutions to the top ten.

**Table 2:** The ten most productive institutions in handgrip strength research by number of publications

Rank	Institution	Country	Publications
1	Universidade de São Paulo	Brazil	273
2	Universidad de Granada	Spain	202
3	Karolinska Institutet	Sweden	175
4	Universidade do Porto	Portugal	141
5	Instituto de Salud Carlos III	Spain	137
6	Universidade Federal de Santa Catarina	Brazil	120
7	Københavns Universitet	Denmark	120
8	University of Melbourne	Australia	115
9	Universidad Pública de Navarra (UPNA)	Spain	104
10	Vrije Universiteit Amsterdam	Netherlands	103

All data are derived from the Scopus database.

**Fig. 4:**
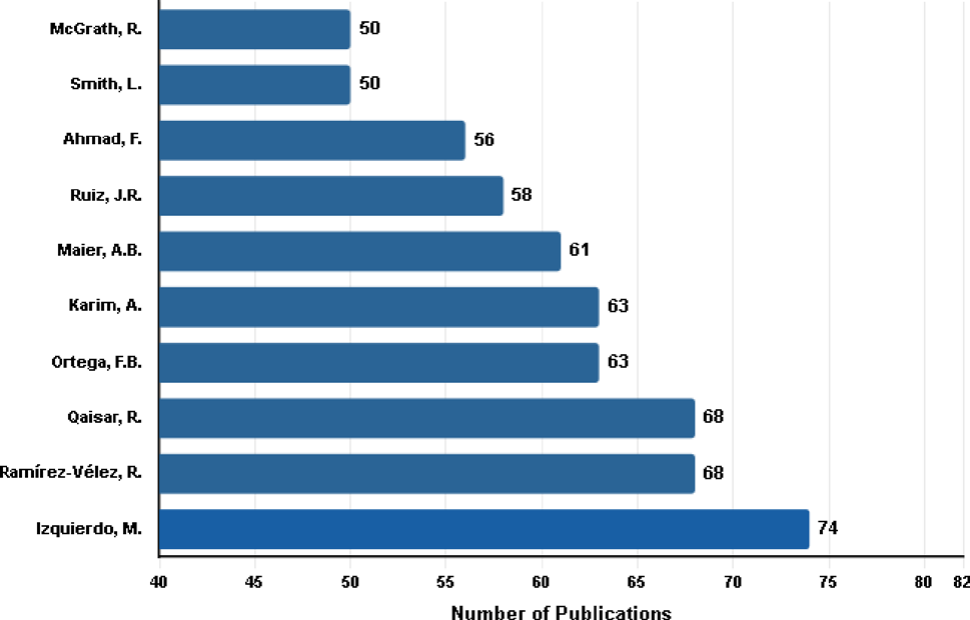
The ten most productive authors by number of publications

The ten most prolific authors in the field collectively produced 611 publications. Izquierdo, M. ranked first with 74 publications, followed by Ramírez-Vélez, R. and Qaisar, R., each with 68 publications. Ortega, F.B., and Karim, A. each contributed 63 publications. Smith, L., and McGrath, R. shared the tenth position, each with 50 publications (Fig. 4).

The five leading funding sponsors collectively supported 1,858 publications. The Coordenação de Aperfeiçoamento de Pessoal de Nível Superior (Brazil) provided funding for the highest number of publications (*n* = 451), followed by the Japan Society for the Promotion of Science (Japan, *n* = 395) and the Conselho Nacional de Desenvolvimento Científico e Tecnológico (Brazil, *n* = 389). The National Institutes of Health (United States, *n* = 338) and the National Natural Science Foundation of China (*n* = 285) ranked fourth and fifth, respectively. Brazil was the most represented country among the leading funding bodies, accounting for two of the top five sponsors (Table 3).

**Table 3:** The five most influential funding sponsors

Rank	Funding sponsor	Country	Publications
1	Coordenação de Aperfeiçoamento de Pessoal de Nível Superior	Brazil	451
2	Japan Society for the Promotion of Science	Japan	395
3	Conselho Nacional de Desenvolvimento Científico e Tecnológico	Brazil	389
4	National Institutes of Health	United States	338
5	National Natural Science Foundation of China	China	285

All data are derived from the Scopus database.

Keyword analysis identified 160 unique terms in the literature. The most frequent keywords were “human” (*n* = 10,428), “female” (*n* = 8,633), and “male” (*n* = 8,584), followed by “humans” (*n* = 8,391) and “article” (*n* = 7,800). Field-specific terms such as “hand strength” (*n* = 7,074) and “grip strength” (*n* = 6,225) ranked sixth and seventh. “Sarcopenia” ranked 17th (*n* = 2,650), and muscle-related terms including “skeletal muscle,” “muscle mass,” and “body composition” were also common (Table 4).

**Table 4:** The twenty most frequently appearing keywords in handgrip strength research

Rank	Keyword	Frequency (number)
1	Human	10,428
2	Female	8,633
3	Male	8,584
4	Humans	8,391
5	Article	7,800
6	Hand Strength	7,074
7	Grip Strength	6,225
8	Adult	6,156
9	Aged	5,897
10	Controlled Study	5,177
11	Muscle Strength	4,210
12	Major Clinical Study	4,113
13	Middle Aged	4,019
14	Physiology	3,701
15	Body Mass	2,928
16	Cross-sectional Study	2,881
17	Sarcopenia	2,650
18	Hand Grip	2,546
19	Exercise	2,424
20	Cross-sectional Studies	2,113

All data are derived from the Scopus database.

The ten most cited publications in this field have accumulated 15,099 citations, demonstrating the breadth and scientific impact of the research. The most cited article is the 2020 consensus update by Chen et al. on sarcopenia diagnosis and treatment, published in the *Journal of the American Medical Directors Association*, which received 5,583 citations. The same research group’s earlier 2014 consensus report from the Asian Working Group for Sarcopenia ranked second with 3,524 citations, also published in the same journal, emphasizing the central role of Asian scholars in shaping the diagnostic framework for sarcopenia. Lauretani et al. (2003) ranked third with 1,567 citations for their influential work on age-associated skeletal muscle changes published in the *Journal of Applied Physiology*. Blake et al. (1988) ranked fourth with 1,071 citations for their foundational study on falls among elderly people at home, illustrating that highly cited works in this field span several decades. The remaining six articles each received between 616 and 844 citations, covering topics such as the global prevalence of sarcopenia, associations between grip strength and cardiovascular or cancer outcomes, physical activity interventions for cancer survivors, and field-based fitness assessments in young people. Overall, the top ten cited articles were published between 1988 and 2020 across eight distinct journals, highlighting the multidisciplinary nature of the literature (Table 5).

**Table 5:** The ten most cited publications in handgrip strength research

Rank	Title	Authors	Journal	Year	Citations
1	Asian Working Group for Sarcopenia: 2019 Consensus Update on Sarcopenia Diagnosis and Treatment	Chen, L.-K. et al.	*Journal of the American Medical Directors Association*	2020	5583
2	Sarcopenia in Asia: Consensus report of the Asian working group for sarcopenia	Chen, L.-K. et al.	*Journal of the American Medical Directors Association*	2014	3524
3	Age-associated changes in skeletal muscles and their effect on mobility: An operational diagnosis of sarcopenia	Lauretani, F. et al.	*Journal of Applied Physiology*	2003	1567
4	Falls by elderly people at home: Prevalence and associated factors	Blake, A.J. et al.	*Age and Ageing*	1988	1071
5	Prevalence of sarcopenia in the world: A systematic review and meta-analysis of general population studies	Shafiee, G. et al.	*Journal of Diabetes and Metabolic Disorders*	2017	844
6	Strength, power and related functional ability of healthy people aged 65–89 years	Skelton, D.A. et al.	*Age and Ageing*	1994	778
7	Physical activity for cancer survivors: Meta-analysis of randomised controlled trials	Fong, D.Y.T. et al.	*BMJ Online*	2012	650
8	Associations of grip strength with cardiovascular, respiratory, and cancer outcomes and all cause mortality: Prospective cohort study of half a million UK Biobank participants	Celis-Morales, C.A. et al.	*BMJ Online*	2018	649
9	Field-based fitness assessment in young people: The ALPHA health-related fitness test battery for children and adolescents	Ruiz, J.R. et al.	*British Journal of Sports Medicine*	2011	621
10	Acquired weakness, handgrip strength, and mortality in critically ill patients	Ali, N.A. et al.	*American Journal of Respiratory and Critical Care Medicine*	2008	616

All data are derived from the Scopus database.

Six retracted publications were identified in the dataset, published between 2021 and 2023 (Table 6). Four articles originated in China, one in Brazil, and one in Indonesia. Citation counts ranged from 0 to 19. Compromised peer review process was the most frequently identified concern, present in four of the six retracted publications.

**Table 6:** Characteristics and retraction reasons of retracted publications identified in handgrip strength research literature

Rank	Title	Country	Year	Citation	Reason
1	High protein diet improves the overall survival in older adults with advanced gastrointestinal cancer	Brazil	2021	13	Substantive errors in reported methods, results, and conclusions; methodological limitations in dietary assessment across different time points; misreporting of sample size (data confirmed as original by authors)
2	Handgrip strength-related factors affecting health outcomes in young adults: association with cardiorespiratory fitness	China	2021	19	Systematic manipulation of the publication process; indicators include discrepancies in research description, data availability, and compromised peer review
3	Effects of Tai Chi softball exercises on physical fitness level and cardiovascular health-related factors among older females	China	2021	1	Systematic manipulation of the publication process; indicators include discrepancies in research description, data availability, and compromised peer review
4	Effects of equine-assistant activity on gross motor coordination in children aged 8 to 10 years	China	2022	0	Compromised peer review process (authors disagreed with retraction)
5	Clinical effect of duloxetine on improving osteoporosis low back pain in older adults	China	2023	0	Compromised editorial handling and peer review process; inappropriate references; concerns regarding scope; loss of confidence in results and conclusions
6	Denosumab’s therapeutic effect for future osteosarcopenia therapy: a systematic review and meta-analysis	Indonesia	2023	8	Methodological flaws affecting study conclusions; retraction requested by authors upon acknowledgment of errors

Country data were determined based on the corresponding author’s affiliated institution.

Retraction reasons were determined based on the retraction notices openly available on PubMed and the respective journal websites.

All data are derived from the Scopus database.

## Discussion

This bibliometric analysis identified a statistically significant upward trend in the scientific literature on HGS from 1944 to the present (B = 8.76; R² = 0.417; *p* < 0.001). The United States, Brazil, and China demonstrated the highest publication volumes, while the most frequently cited studies addressed sarcopenia diagnostic criteria and musculoskeletal epidemiology. Analyses of journals, institutions, authors, and funding sources indicate that research activity was concentrated within specific domains. 

The significant rise in HGS-related publications over the years demonstrates the field’s growing prominence in scientific research. Several factors contribute to this trend. The clarification of sarcopenia diagnostic criteria by international groups such as the European Working Group on Sarcopenia in Older People (EWGSOP) and the Asian Working Group for Sarcopenia (AWGS), along with the inclusion of HGS as a key component, has focused researchers’ attention on this area [24, 25]. Additionally, the incorporation of HGS measurement into clinical guidelines and routine screening protocols has increased academic interest [26, 27]. The global increase in the elderly population and the associated rise in chronic diseases have also highlighted the importance of studying the relationship between muscle function and overall health.

HGS research conducted in 128 countries demonstrates that this subject has become a universal area of clinical interest. The leading position of the United States is expected, as the country maintains a pioneering role in many biomedical fields [28]. This is further supported by the National Institutes of Health, which ranks among the top five funding sponsors in the current analysis. Brazil’s second-place ranking suggests that Latin America’s research capacity in health sciences has improved significantly in recent years [29]. The dual representation of Brazil among both leading institutions and funding bodies further reflects a coordinated national investment in this field. China’s advancement to third place reflects the rapid transformation in the country’s scientific productivity [30]. This growth may result from China’s aging population and health policies that prioritize musculoskeletal research. However, identifying 30 countries as the main active category indicates that research activities remain concentrated in a certain number of countries.

The leading journals by publication volume were multidisciplinary periodicals, including *Nutrients*, *International Journal of Environmental Research* and *Public Health*, and *PLOS One*. This distribution demonstrates that HGS research spans a broad thematic scope, including nutrition science, public health, and general medicine. The presence of *BMC Geriatrics* among these journals further underscores the significance of HGS in clinical research focused on the elderly population. Analysis of document types reveals that original articles represented the majority of the literature.

The prominence of Brazil and Spain among the most productive institutions corresponds with their leading positions in country-based publication analyses, suggesting that their research capacity is underpinned by both publication volume and a robust, sustainable scientific infrastructure. Analysis of funding sources further substantiates this observation. Brazil’s presence at both the institutional and funding levels, with two leading funding organizations and two highly productive universities, indicates a systematic and coordinated national investment policy in HGS research. The presence of Japanese and Chinese funding organizations among the top five further demonstrates Asia’s increasing scientific influence in this field.

The keyword analysis demonstrates that HGS research centers on methodological rigor and measurement standardization, as evidenced by frequent terms such as “hand strength” and “grip strength.” The prominence of “sarcopenia” highlights HGS’s role in diagnosing and evaluating this condition, consistent with the high citation rates of consensus reports on sarcopenia. Additionally, frequent references to “skeletal muscle,” “muscle strength,” “muscle mass,” and “body composition” indicate a primary focus on muscle mass loss, impaired function, and related clinical outcomes.

Most highly cited studies examined diagnostic criteria for sarcopenia and its relationship to clinical outcomes of muscle mass loss. The frequent citation of Blake et al.‘s 1988 study highlights the ongoing relevance of HGS research to core clinical issues, including fall risk and functional independence. Additionally, studies on physical activity interventions, cardiovascular outcomes, and youth fitness assessment demonstrate that HGS is now recognized as a key research parameter across fields such as oncology, cardiology, and sports sciences. In this regard, Fong et al. (2012), ranked seventh with 650 citations, demonstrated the benefits of physical activity interventions for cancer survivors, while Celis-Morales et al. (2018), ranked eighth with 649 citations, reported associations between grip strength and cardiovascular, respiratory, and cancer outcomes in a cohort of half a million participants. The high citation counts of both studies provide robust epidemiological evidence for the expanding clinical utility of HGS across oncology and cardiology (Table 5). Furthermore, survey-based studies indicate that clinicians increasingly recognize the prognostic value of HGS as a practical and informative tool in routine clinical assessment [31].

The identification of peer-review process issues across four articles underscores the need to strengthen editorial control mechanisms in scientific journal publishing. Although most retracted articles originated from China, likely reflecting the country’s significant contribution to global publication volume, a comparative evaluation of retraction patterns in the HGS field with those in other biomedical disciplines may provide valuable insights for future research.

Several limitations of this bibliometric analysis should be noted. First, the study relies solely on data from the Scopus database. Second, restricting the analysis to English-language publications may overlook research in other languages and introduce geographical bias. Third, bibliometric analyses have inherent methodological limitations; citation counts measure quantitative impact but do not reflect methodological quality or clinical significance. Finally, data for 2026 only covers January through April, so findings for that year are incomplete.

## Conclusion

This bibliometric analysis reveals that the scientific literature on HGS has grown significantly from 1944 to the present. With 12,221 publications and contributions from 128 countries, the literature displays a broad global distribution, indicating that HGS research extends beyond sarcopenia and geriatric medicine to become central in fields such as oncology, cardiology, public health, and sports sciences. The leading roles of the United States, Brazil, and China underscore the field’s international scope, while the dual representation of Spain and Brazil at both institutional and funding levels suggests a coordinated, sustained investment strategy. The predominance of consensus reports among the most cited studies, particularly those addressing the diagnosis and treatment of sarcopenia, confirms that HGS has established clinical value. As the global population ages and chronic diseases become more prevalent, scientific interest in HGS is likely to grow. To advance the field, it is important to increase the involvement of low- and middle-income countries, strengthen international collaboration, and promote the use of standard measurement protocols.

## Data Availability

Raw data can be provided upon request.

## References

[1] National Library of Medicine (1995) MeSH descriptor data: hand strength [D018737]. U.S. National library of medicine. https://www.ncbi.nlm.nih.gov/mesh/68018737. Accessed 26 April 2026

[2] Szaflik P, Zadoń H, Michnik R, Nowakowska-Lipiec K (2025) Handgrip strength as an indicator of overall strength and functional performance—systematic review. Appl Sci (Basel) 15:1847. 10.3390/app15041847

[3] Kothari R, Johny MP, Mistry D, Irfana M, Yogesh S, Vemparala SS (2024) Grip to health: unlocking the clinical potential of isometric hand grip strength - a narrative review. J Pharm Bioallied Sci 16:S3102–S3104. 10.4103/jpbs.jpbs_1040_24 39926740 10.4103/jpbs.jpbs_1040_24 PMC11805275

[4] Vaishya R, Misra A, Vaish A, Ursino N, D’Ambrosi R (2024) Hand grip strength as a proposed new vital sign of health: a narrative review of evidences. J Health Popul Nutr 43:7. 10.1186/s41043-024-00500-y 38195493 10.1186/s41043-024-00500-y PMC10777545

[5] Quattrocchi A, Garufi G, Gugliandolo G, De Marchis C, Collufio D, Cardali SM, Donato N (2024) Handgrip strength in health applications: a review of the measurement methodologies and influencing factors. Sensors (Basel) 24:5100. 10.3390/s24165100 39204796 10.3390/s24165100 PMC11359434

[6] Fedorchenko Y, Imanbayeva N, Khojakulova U, Assylbek MI, Zimba O (2025) Sarcopenia in rheumatic and musculoskeletal diseases: pathophysiology, diagnosis, and management. Rheumatol Int 45:266. 10.1007/s00296-025-06027-7 41196407 10.1007/s00296-025-06027-7

[7] Hanna Deschamps E, Herrmann FR, De Macedo Ferreira D, Silva M, Graf CE, Mendes A (2025) Two methods of handgrip strength assessment in sarcopenia evaluation: associations with in-hospital mortality in older adults. Clin Interv Aging 20:1619–1634. 10.2147/CIA.S529761 40995375 10.2147/CIA.S529761 PMC12456314

[8] Fedorchenko Y, Khojakulova U, Zimba O, Kocyigit BF (2026) Handgrip strength testing in rheumatic diseases. Rheumatol Int 46:82. 10.1007/s00296-026-06111-6 42053814 10.1007/s00296-026-06111-6 PMC13128709

[9] Lee SH, Gong HS (2020) Measurement and interpretation of handgrip strength for research on sarcopenia and osteoporosis. J Bone Metab 27:85–96. 10.11005/jbm.2020.27.2.85 32572369 10.11005/jbm.2020.27.2.85 PMC7297622

[10] Xiao M, Lu Y, Li H, Zhao Z (2024) Association between handgrip strength and mortality of patients with coronary artery disease: a meta-analysis. Clin Cardiol 47:e24322. 10.1002/clc.24322 39051437 10.1002/clc.24322 PMC11270052

[11] Žura N, Vukorepa M, Jurak I et al (2021) Decrease in handgrip strength in rheumatoid arthritis (RA): is there a sex-related difference? Rheumatol Int 41:1795–1802. 10.1007/s00296-021-04959-4 34319448 10.1007/s00296-021-04959-4

[12] Moschou D, Krikelis M, Georgakopoulos C, Mole E, Chronopoulos E, Tournis S, Mavragani C, Makris K, Dontas I, Gazi S (2024) Prevalence and factors associated with sarcopenia in post-menopausal women with rheumatoid arthritis. Mediterr J Rheumatol 35:438–447. 10.31138/mjr.260323.paf 39463868 10.31138/mjr.260323.paf PMC11500114

[13] Huerta-Uribe N, Hormazábal-Aguayo I, Muñoz-Pardeza J et al (2024) Handgrip strength, cardiometabolic risk and body composition in youth with type 1 diabetes: the Diactive-1 Cohort Study. BMJ Open Sport Exerc Med 10:e002177. 10.1136/bmjsem-2024-002177 39650570 10.1136/bmjsem-2024-002177 PMC11624725

[14] Sánchez-Delgado JC, Cohen DD, Camacho-López PA et al (2023) Handgrip strength is associated with specific aspects of vascular function in individuals with metabolic syndrome. Biomedicines 11:2435. 10.3390/biomedicines11092435 37760876 10.3390/biomedicines11092435 PMC10525985

[15] Lee SR, Cho YH, Park EJ, Lee Y, In Choi J, Kwon RJ, Son SM, Lee SY (2024) The association between reproductive period and handgrip strength in postmenopausal women: a nationwide cross-sectional study. Menopause 31:26–32. 10.1097/GME.0000000000002283 38016167 10.1097/GME.0000000000002283

[16] Kim SW, Lee HA, Cho EH (2012) Low handgrip strength is associated with low bone mineral density and fragility fractures in postmenopausal healthy Korean women. J Korean Med Sci 27:744–747. 10.3346/jkms.2012.27.7.744 22787368 10.3346/jkms.2012.27.7.744 PMC3390721

[17] Gasparyan AY, Ayvazyan L, Blackmore H, Kitas GD (2011) Writing a narrative biomedical review: considerations for authors, peer reviewers, and editors. Rheumatol Int 31:1409–1417. 10.1007/s00296-011-1999-3 21800117 10.1007/s00296-011-1999-3

[18] Fedorchenko Y, Zimba O (2023) Comorbidities in the COVID-19 pandemic: Scopus-based bibliometric analysis. J Korean Med Sci 38:e93. 10.3346/jkms.2023.38.e93 36942396 10.3346/jkms.2023.38.e93 PMC10027540

[19] Kocyigit BF, Akyol A (2021) Bibliometric analysis of publication activity in the field of Familial Mediterranean Fever in 2010–2019: a Scopus-based study. Rheumatol Int 41:2015–2023. 10.1007/s00296-021-04988-z 34499195 10.1007/s00296-021-04988-z

[20] Zhao X, Ye R, Zhao L, Lin Y, Huang W, He X, Lian F, Tong X (2015) Worldwide research productivity in the field of endocrinology and metabolism: a bibliometric analysis. Endokrynol Pol 66:434–442. 10.5603/EP.2015.0054 26457499 10.5603/EP.2015.0054

[21] Akyol A, Kocyigit BF (2021) Ankylosing Spondylitis rehabilitation publications and the global productivity: a Web of Science-based bibliometric analysis (2000–2019). Rheumatol Int 41:2007–2014. 10.1007/s00296-021-04836-0 33797569 10.1007/s00296-021-04836-0

[22] Stavale R, Ferreira GI, Galvão JAM, Zicker F, Novaes MRCG, Oliveira CM et al (2019) Research misconduct in health and life sciences research: a systematic review of retracted literature from Brazilian institutions. PLoS One 14:e0214272 30986211 10.1371/journal.pone.0214272 PMC6464327

[23] Kocyigit BF, Zhakipbekov K, Yessirkepov M (2025) Analysis of retracted publications on methotrexate. J Korean Med Sci 40:e243 41025340 10.3346/jkms.2025.40.e243 PMC12480959

[24] Cruz-Jentoft AJ, Bahat G, Bauer J et al (2019) Sarcopenia: revised European consensus on definition and diagnosis. Age Ageing 48:16–31. 10.1093/ageing/afy169 30312372 10.1093/ageing/afy169 PMC6322506

[25] Chen LK, Woo J, Assantachai P et al (2020) Asian Working Group for Sarcopenia: 2019 consensus update on sarcopenia diagnosis and treatment. J Am Med Dir Assoc 21:300–307. 10.1016/j.jamda.2019.12.012 32033882 10.1016/j.jamda.2019.12.012

[26] Bohannon RW (2019) Grip strength: an indispensable biomarker for older adults. Clin Interv Aging 14:1681–1691. 10.2147/CIA.S194543 31631989 10.2147/CIA.S194543 PMC6778477

[27] Roberts HC, Denison HJ, Martin HJ, Patel HP, Syddall H, Cooper C, Sayer AA (2011) A review of the measurement of grip strength in clinical and epidemiological studies: towards a standardised approach. Age Ageing 40:423–429. 10.1093/ageing/afr051 21624928 10.1093/ageing/afr051

[28] Yerlanova D, Mukhamediyarov M, Zimba O, Korkosz M, Kocyigit BF (2026) Systemic lupus erythematosus-related infections in pregnancy: a cross-sectional bibliometric analysis. Rheumatol Int 46:63. 10.1007/s00296-026-06086-4 41870562 10.1007/s00296-026-06086-4 PMC13009124

[29] Zacca-González G, Chinchilla-Rodríguez Z, Vargas-Quesada B, de Moya-Anegón F (2014) Bibliometric analysis of regional Latin America’s scientific output in public health through SCImago Journal & Country Rank. BMC Public Health 14:632. 10.1186/1471-2458-14-632 24950735 10.1186/1471-2458-14-632 PMC4094685

[30] Owens B (2024) China’s research clout leads to growth in homegrown science publishing. Nature 630:S2–S4. 10.1038/d41586-024-01596-2 38840022 10.1038/d41586-024-01596-2

[31] Myles L, Massy-Westropp N, Barnett F (2024) The how and why of handgrip strength assessment. Br J Occup Ther 87:321–328. 10.1177/03080226231208409 40337531 10.1177/03080226231208409 PMC12033516

